# Role of Vitamin D in the Prevention of Pancreatic Cancer

**DOI:** 10.1155/2010/721365

**Published:** 2011-01-09

**Authors:** Pubudu Bulathsinghala, Kostas N. Syrigos, Muhammad W. Saif

**Affiliations:** ^1^Department of Internal Medicine, Danbury Hospital, 24 Hospital Avenue, Danbury, CT 06810, USA; ^2^Oncology Unit, 3rd Department of Medicine, Sotiria General Hospital, Athens School of Medicine, Mesogion 152, 11527 Athens, Greece; ^3^GI Oncology Section of the Division of Hematology/Oncology, Herbert Irving Comprehensive Cancer Center, Milstein Hospital, Clinical Medicine, Columbia University College of Physicians and Surgeons, Suite 6-435, 177 Fort Washington Avenue, NY 10032, USA

## Abstract

Pancreatic cancer is a malignancy of poor prognosis which is mostly diagnosed at advanced stages. Current treatment modalities are very limited creating great interest for novel preventive and therapeutic options. Vitamin D seems to have a protective effect against pancreatic cancer by participating in numerous proapoptotic, antiangiogenic, anti-inflammatory, prodifferentiating, and immunomodulating mechanisms. 25-hydroxyvitamin D [25(OH)D] serum concentrations are currently the best indicator of vitamin D status. There are three main sources of vitamin D: sun exposure, diet,and dietary supplements. Sun exposure has been associated with lower incidence of pancreatic cancer in ecological studies. Increased vitamin D levels seem to protect against pancreatic cancer, but caution is needed as excessive dietary intake may have opposite results. Future studies will verify the role of vitamin D in the prevention and therapy of pancreatic cancer and will lead to guidelines on adequate sun exposure and vitamin D dietary intake.

## 1. Introduction

### 1.1. Epidemiology

Pancreatic cancer exhibits some of the lowest overall survival rates in oncology, and its incidence has been underestimated for years. Approximately 6% of all cancer-related deaths are associated with pancreatic cancer and around 32,000 Americans are diagnosed and die from this disease annually [1]. It is the fourth leading cause of cancer mortality in both men and women. One-year and 5-year survival rates are estimated at 24% and 4.3%, respectively [2]. One of the main reasons for this dismal prognosis is the lack of an effective screening method as pancreatic cancer is difficult to diagnose in its early disease stages. At time of diagnosis, 52% of patients have distant metastases and in 26% of cases, the disease has spread locoregionally [2]. 

Pancreatic cancer has a higher prevalence among men and African-Americans. Results from several epidemiologic studies have suggested that several environmental factors may be associated with developing pancreatic cancer, but tobacco smoking was the only established risk factor [3]. It was shown that a larger waist circumference and waist-to-hip ratio were associated with a statistically significant increased risk of developing pancreatic cancer, although greater body mass index and lack of total physical activity were not identified as risk factors [3]. According to Patel et al., obesity and central adiposity are correlated to increased pancreatic cancer risk [4]. Diabetes mellitus was associated with greater pancreatic cancer incidence [5, 6], although there is limited data supporting the theory that sugars or sweets are pathogenetically implicated [7]. Finally, high red and processed meat intake was linked to elevated risk but this is probably secondary to carcinogenic substances used during meat processing.

### 1.2. Current Treatment Modalities

The only existing pancreatic cancer treatment which offers the potential of cure is surgical resection. Nevertheless, as stated above, most patients are diagnosed at advanced stage and are not likely candidates for surgical therapy [2].

Despite continuing research, limited progress has been made in the treatment of advanced pancreatic cancer. For over a decade, gemcitabine was the acceptable standard treatment, but its use as monotherapy in advanced and metastatic stages of pancreatic cancer has been in question as only small benefit has been shown. Multiple gemcitabine-based therapeutical regimens have been studied (i.e. gemcitabine combined with molecular targeting agents, farnesyltransferase inhibitors, and metalloproteinase inhibitors) [2]. The combination of gemcitabine plus erlotinib is the only one that seems to prolong survival [8].

Therefore, treatment for patients with advanced pancreatic cancer is primarily palliative. Initial monotherapy with 5-fluorouracil (5-FU) has been widely administered, but it did not demonstrate any benefit for overall quality of life or survival. Response rates were less than 10% [9]. Clinical trials with chemoradiation and multiple chemotherapeutic regimens (doxorubicin or doxorubicin/mitomycin, cisplatin) alongside 5-FU failed to prolong overall survival, although response rates were better with some of the regimens. Increased toxicity was another reason that these regimens were not explored further [10–12].

### 1.3. Genetic Basis for Pancreatic Cancer

The genetic basis of pancreatic cancer has been extensively studied. Mutation or silencing of p53, p1, and DPC4/s mad4 genes is associated with pancreatic cancer, but K-*ras* mutations (in codons 12, 13, and 16) are the most frequently noticed mutations [13]. K-*ras* mutations have also been found to poor prognostic factors for patients who have undergone surgery and adjuvant chemoradiation. It should also be noted that in a preclinical study Fleming et al showed that certain deleting K-*ras* mutations may result in altering cancerous behavior of pancreatic tumor cells [14].

## 2. Vitamin D Is a Protective Factor

Due to the fact that pancreatic cancer carries one of highest mortality rates and is very resistant to treatment, there are efforts to find protective factors. Vitamin D seems to prevent not only pancreatic cancer, but also colon, ovary, and breast cancer [15–18]. According to a most recent epidemiological analysis by Garland et al, raising serum levels of vitamin D would prevent 58000 new cases of breast cancer and 49000 of new cases of colorectal cancer each year [19].

### 2.1. Sources of Vitamin D

There are three main vitamin D sources for humans: sunlight exposure (ultraviolet B radiation: UVB), diet, and dietary supplements [20, 21]. Sunlight irradation is the most important, and early-life exposure has already been associated with decreased cancer risk in several malignancies, such as prostate cancer [22–24]. Brief body exposure directly to direct sunlight during summer is all that is required to make enough cholecalciferol (vitamin D_3_) for the daily vitamin D requirements. During sun exposure, UVB radiation (wavelength of 290 to 315 nm) pierces through the skin and converts 7-dehydrocholesterol (provitamin D3) in the skin to previtamin D3. Since previtamin D3 is very unstable, it is rapidly transformed to its more stable form of vitamin D3, which is then absorbed across the subcutaneous capillary bed to enter general circulation [20, 25]. Mohr et al. (2010) confirmed earlier findings that the incidence of pancreatic cancer was higher in countries with lower UVB irraidiance [26]. There is an ever growing number of ecological studies regarding cancer incidence that corroborate the inverse correlation of pancreatic cancer to solar UVB exposure in Western and in Asian countries [27–34]. Ecological studies are very helpful when examining such a topic, as much of the risk for cancer occurs with early-life effects in the first 20 years of life, starting with conception [35]. A recent study used Hill's criteria of causality to assess the value of UVB and vitamin D in reducing cancer risk and concluded that results for several cancers satisfy these criteria [36, 37]. Vitamin D intoxication secondary to prolonged sun exposure is not observed, as excess previtamin D3 and vitamin D3 readily absorb sunlight and are converted into inactive photoproducts [38]. Thus, vitamin D intoxication through sunlight has never been reported in life guards, who experience excessive sun exposure [38]. There are public health concerns since excess ultraviolet radiation exposure is implicated in skin cancer, development of cataract, premature photoageing and skin hypersensitivity when certain drugs are concomitantly used, even with a short amount of time of sun exposure [39]. It is worth noting that vitamin D produced which during the sunny months (spring, summer and fall) is stored in the body fat but it is not known whether it can be actually used in winter months, unless, maybe, in case of significant adipose tissue loss [40]. Unfortunately, vitamin D production through sunlight exposure is often not sufficient for people with dark skin, urban areas as well as for those largely confined indoors [39, 41, 42]. Furthermore, latitude plays an important role because in temperate zones (23.5°–66.5°), people are unable to synthesize vitamin D for 1 month of the year due to insufficient UVB and those nearer to the poles (>66.5°) do not get enough UVB radiation for most of the year [43, 44].

In absence of required sunlight exposure, vitamin D can be obtained from dietary sources or supplements [39]. Only few foods are known to naturally contain considerable amounts of vitamin D: oily fish such as sardines, salmon, and mackerel and fish oils such as cod liver oil [40, 45]. Certain foods, such as milk, orange juice, bread, and cereal, are fortified with vitamin D [46–48]. There are two forms of supplemental vitamin D, D2, and D3 [45]. Ergosterol is produced in plants and yeast, whereas 7-dehydrocholesterol, which is its immediate precursor in the cholesterol biosynthetic pathway, is produced in animals [40, 45]. UVB sunlight radiation converts ergosterol and 7-dehydrocholesterol, into vitamin D2 (ergocalciferol) and D3 (cholecalciferol), respectively [40, 45]. Although some believe D3 is more potent than D2 [49], this has not been confirmed by the literature [50].

### 2.2. Metabolism of Vitamin D

Vitamin D (both D_2_ and D3 forms) is converted to 25-hydroxycholecalciferol [25(OH)D-also known as calcidiol] in the liver. This form is then further hydroxylated to 1,25-dihydroxycholecalciferol [1,25(OH)_2_D-also known as calcitriol] in the kidneys [20, 21]. 25-hydroxycholecalciferol is used to measure the vitamin D levels, although 1,25-dihydroxycholecalciferol is the active form [20, 21]. Therefore, hereon in this review, the term vitamin D will refer to 25-hydroxycholecalciferol, unless otherwise stated. High-affinity receptors in the nucleus help the active compound regulate gene transcription in many cells [21]. They are found not only in target organs (gut, bone, kidney, and parathyroid) [51], but also in many other tissues, such as brain, breast, colon, heart, pancreas, prostate, skin, and the immune system [52]. The rate-limiting step of the vitamin D conversion pathway is interesting: the vitamin D 1-*α*-hydroxylase enzyme converts 25(OH)D to 1,25(OH)_2_D and was initially thought to exist only in kidneys which regulate the systemic levels of vitamin D. It has been recently found that this enzyme is expressed in many other organs as well, such as the pancreas. This locally produced calcitriol does not enter systemic circulation and therefore does not affect calcium metabolism. It remains in the region and offers protection to the cells that produce it and cells nearby [52].

### 2.3. Protective Action of Vitamin D and Probable Molecular Mechanisms

The importance of vitamin D in cancer prevention was originally derived from epidemiological data that associated season of diagnosis and sun exposure with cancer incidence and mortality [53–62]. From these observational studies, it was obvious that sun exposure was inversely correlated to cancer incidence and mortality. Protective features of solar radiation exposure have been attributed to elevated serum levels of 25(OH)D.

This hypothesis was later supported by dietary [58, 63] and mechanistic studies [64–66]. It has been shown that vitamin D not only has a protective but also a therapeutic effect on many malignancies [67–72]. These actions of vitamin D are mediated by a number of proapoptotic, antiangiogenic, anti-inflammatory, prodifferentiating, and immunomodulating mechanisms [16, 73, 74]. Despite theoretical concerns, evidence-based studies have shown that vitamin D has no adverse effect on chemotherapy or radiation therapy [75]. More over, there seems to be synergy with chemotherapy and radiotherapy. The role of vitamin D as an anticancer agent is just starting to be explored; however, initial results are promising [76–78]. A recent phase II study showed that high-dose calcitriol with docetaxel may increase time to progression in patients with incurable pancreatic cancer when compared to docetaxel monotherapy [79]. Paricalcitol is a less calcemic vitamin D analogue which has been found to be in vitro and in vivo effective in inhibiting tumor growth in vitro and in vivo, via upregulation of p21 and p27 tumor suppressor genes. It constitutes an attractive novel therapy for pancreatic cancer [74].

The active form of vitamin D, 1,25(OH)_2_D, or calcitriol is as a steroid hormone involved in molecular mechanisms that prevent cellular malignant transformation. Some of the vital cellular actions of vitamin D are involved in intracellular signaling pathways [66], cell cycle and cellular growth [80, 81], differentiation [82, 83], adhesion [84], and apoptosis [85, 86]. Calcitriol mediates all these actions by binding to a nuclear receptor known as vitamin D receptor (VDR), which is expressed in many cell types [87]. It enters the cellular cytoplasm and binds to nuclear VDR. This complex migrates to the nucleus, binds to vitamin D-responsive elements (VDREs), and activates promoters of responsive target genes. Based on the type of complex formed, calcitriol regulates initiation or suppression of gene expression [84]. A brief account of the effects of vitamin D on cell division, differentiation, adhesion, and apoptosis is summarized below.

1. Cellular division and differentiation.
  - Vitamin D regulates the levels of p21 and p27, which in turn control the cell cycle [88].
  - Cyclin D activates cyclin-dependant kinases (CDKs) in order to increase transcription of genes controlling the transition from G_1_ to S phase. Calcitriol binds to cyclin D and inhibits this transition [88].
  - The accuracy of DNA replication and DNA repair is controlled by cell cycle surveillance mechanisms. Tumor suppressor gene p53 is activated in case of DNA damage and increases p21 levels; therefore, CDKs are inhibited and cell cycle arrest is induced. Vitamin D is involved in this process as it regulates p21 and p53 levels [89, 90].
  - Calcitriol increases expression of BRCA-1 and -2 tumor suppressor genes contributing in the DNA repair mechanism [91].
2. Cell adhesion: when a cancer cell loses its adhesion features, it acquires metastatic potential. Calcitriol increases E-cadherin and changes the location of *β*-catenin, reducing intracellular mRNA concentration of c-myc [69].
3. Cell death (apoptosis): caspases mediate apoptosis and are activated by p53, which is increased in DNA damage. Calcitriol upregulates genes involved in apoptosis by regulating p53 levels [92, 93].

Vitamin D is implicated in other molecular pathways as well. It is known to decrease vascular growth factor (VEGF), which is essential in tumor angiogenesis [45]. VDR growth control has been recently found to vary considerably between different malignancies suggesting tissue-specificity of the above vitamin D dependent pathways [94]. Finally, vitamin-D-binding-protein (DBP) is considered to be deglycosylated in cancer patients causing inability to activate macrophages. More specifically, Gc protein-derived macrophage activating factor (GcMAF) is a naturally derived form of human DBP with a single terminal O-linked GalNac sugar residue and plasma levels of O-linked trisaccharide glycosylated DBP have been found associated with GcMAF precursor activity. Studies in cancer patients have shown that *α*-N-acetylgalactosaminidase (nagalase) activity is inversely correlated to this precursor activity, and this could explain why cancer patients are relatively unable to activate macrophages. However, reports on this subject have been conflicting [95].

Recent studies have demonstrated the role of vitamin D in pancreatic cancer. It reduces the risk for pancreatic cancer by regulating cell cycle and differentiation [96]. Vitamin D 1-*α*-hydroxylase enzyme levels were found elevated in malignant pancreatic cells, suggesting that vitamin D is a protective factor [96]. EB1089, an calcitriol analogue with low calcemic activity, is a potent inhibitor of parathyroid hormone-related peptide (PTHRP) production *in vitro* and has shown protective features concerning proliferation and differentiation of pancreatic cancer cells [97] and xenografted malignant cells [98].

### 2.4. Role of Dietary Vitamin D

Although several environmental factors have been studied, only smoking has been found to be strongly associated with pancreatic cancer. Serum levels of vitamin D have been extensively studied in several malignancies. Prediagnostic levels were not always associated with incidence [99] but numerous studies have shown that higher serum levels at diagnosis are correlated with increased survival [100–104]. The literature concerning the role of diet in the development of pancreatic cancer is minimal. One study that examined dietary vitamin D intake and pancreatic cancer was not able to show an association [105]. However, this could be attributed to the fact that the median reported dietary intake of vitamin D in this study was about half of the U.S. defined required daily intake of vitamin D. Skinner et al. suggested a role of dietary vitamin D in pancreatic cancer oncogenesis and prevention. They monitored dietary vitamin D intake and pancreatic cancer incidence over a period of 16 years. They used a standardized 131-item semiquantitative food-frequency questionnaire to monitor vitamin D intake. The final analysis of their prospective cohort study showed that participants that consumed ≥600 IU/d of dietary vitamin D had 41% lower risk for pancreatic cancer when compared to those consuming <150 IU/d [63]. The results of the American Institute for Cancer Research Report [106] as well as those of the most recent systematic review of the Tufts Practice Center sponsored by the Institute of Medicine and the World Health Organization [107] have been valuable regarding vitamin D-calcium interactions and Dietary Reference Intake (DRI) recommended values are concerned. Nonhypercalcemic analogues of the active form of cholecalciferol may offer chemoprevention against gastrointestinal cancer by tightly regulating cell differentiation.

One of the major limitations in the analyses of these studies is the lack of an accurate nutrient database in order to assess vitamin D levels in dietary, supplemental and pharmaceutical sources. A widely accepted reference is the United States Department of Agriculture (USDA) National Nutrient Database, which is currently being revised. The new version should be compared to the current one in order to assess, whether the association which found between vitamin D and pancreatic cancer actually exists.

### 2.5. Adequate Levels and Sources of Vitamin D

There is a continuous debate concerning the adequate level of vitamin D in the human body. Even though there is no established consensus, 25(OH)D levels lower than 20 ng per liter (50 nmol per liter) are usually defined as vitamin D deficiency [108, 109]. 25(OH)D levels ranging from 21–29 ng (52–72 nmol per liter) are considered as relatively insufficient and levels greater than 30 ng (75 nmol per liter) can be accepted sufficient [110, 111]. Finally, experts consider levels exceeding 150 ng per liter (374 nmol per liter) as vitamin D intoxication [45]. Unfortunately, all these reference levels are based on myoskeletal studies and the required levels for nonskeletal functions might be different. In malignancies, vitamin D levels are high both systemically as well as locally therefore, the optimum level is expected to be higher. Garland et al. assume that this level will be at least as high as 40 to 50 ng/mL (100–125 nmol/L), and some experts consider these levels as adequate to prevent malignancies [16, 73]. However, opinions vary as cohorts have contradictory results on this subject [112, 113]. A most recent study reported that greatly increased vitamin D concentrations were associated with a statistically significant 2-fold increase in overall pancreatic cancer risk therefore, recommendations on increasing vitamin D intake should be thoroughly considered [114]. 

Regarding the distribution of pancreatic cancer risk, there have been two studies suggesting a U-shaped serum 25(OH)D level dose-disease relation [115, 116]. These findings have not been reproduced by other studies but the differences may be attributed to differences in the vitamin D pancreatic risk association between men and women [63], smokers and nonsmokers [117], and high and low residential UVB exposure [115]. A critical approach by Grant et al. (2009) [117] supports that data on U-shaped distribution are too limited to be used as basis for public health policies. The review of the International Agency for Research on Cancer also noted the need to further assess the potential for U-shaped association between vitamin D status and risk of pancreatic cancer [116].

In order to achieve serum levels of 50 ng/ml, oral supplementation of 4000 IU/day vitamin D is recommended or a supplementation of 2000 IU/day plus 12 minutes of daily sun exposure of 50% of the body surface area [16]. These doses are very unlikely to cause intoxication; however, it is advisable to monitor vitamin D levels every 3 months. Finally, optimum and adequate levels of vitamin D need to be defined for each disease category and recommendations are still under debate. Even though the daily dietary requirements vary with age and sun exposure, the currently accepted vitamin D daily dietary intake is 200–600 IU/day [16].

### 2.6. Assessment of Vitamin D Levels

Serum 25(OH)D is used to estimate vitamin D body levels. However, as most tissues express vitamin D 1-*α*-hydroxylase (CYP27B1), the active form of vitamin D, 1,25(OH) vitamin D, may be increased locally. Therefore, based on the systemic levels of 25(OH)D, we are likely to underestimate the local active form concentration, which is the most important factor for tumor suppression [16]. Depending on sun exposure, 25(OH)D levels also tend to fluctuate around the year. Daily levels do not fluctuate significantly within the same season, but they are known to vary greatly between seasons. Consequently, it should be noted that serum levels reflect dietary intake as well as sun exposure and they do not represent long-term exposure, which is of interest in epidemiological studies. Serum vitamin D levels are also thought to be influenced by genotype (VDR polymorphisms, CYP27A1, CYP27B1, CYP24A1 variant alleles) as the latter affects vitamin D transport and metabolism [73, 118–120]. The best method of estimating vitamin D serum levels is yet to be defined.

## 3. Conclusion

Recent literature has provided plenty of information concerning the preventive and therapeutic role of vitamin D in many malignancies, including pancreatic cancer. Daily doses needed to achieve protective levels have not been determined yet, and it is possible that levels vary between cancer types and stages. Increase vitamin D levels seem to protect against pancreatic cancer but caution is needed as greatly increased dietary intake may have opposite results. No accurate nutrient database has been established yet; therefore, interpretation of data should be done with caution. Better assessment of sun exposure in the future as well as better food compositional data may help clarify whether the association between vitamin D and pancreatic cancer actually exists. Ongoing clinical trials will assist in verifying the role of vitamin D in the prevention and therapy of pancreatic cancer and in clarifying the molecular pathways implicated. Future studies will lead to guidelines concerning adequate sun exposure and vitamin D dietary intake.
